# Carbapenem triggers dissemination of chromosomally integrated carbapenemase genes via conjugative plasmids in *Escherichia coli*

**DOI:** 10.1128/msystems.01275-22

**Published:** 2023-06-05

**Authors:** Ryuichiro Abe, Yukihiro Akeda, Yo Sugawara, Yuki Matsumoto, Daisuke Motooka, Tetsuya Iida, Shigeyuki Hamada

**Affiliations:** 1 Japan-Thailand Research Collaboration Center on Emerging and Re-Emerging Infections, Research Institute for Microbial Diseases, Osaka University, Osaka, Japan; 2 Department of Bacterial Infections, Research Institute for Microbial Diseases, Osaka University, Osaka, Japan; 3 Department of Bacteriology Ⅰ, National Institute of Infectious Diseases, Tokyo, Japan; 4 Division of Infection Control and Prevention, Graduate School of Medicine, Osaka University, Osaka, Japan; 5 Antimicrobial Resistance Research Center, National Institute of Infectious Diseases, Tokyo, Japan; 6 Department of Infection Metagenomics, Research Institute for Microbial Diseases, Osaka University, Osaka, Japan; 7 Center for Infectious Disease Education and Research, Osaka University, Osaka, Japan; Lebanese American University, Byblos, Lebanon

**Keywords:** chromosomal integration, plasmid excitation, carbapenemase, carbapenem resistance, CRE, bacterial community, antimicrobial resistance enhancement, amplified genes, plasmid elimination, plasmid maintenance

## Abstract

Epidemiological surveys have shown that carbapenem resistance is mainly transmitted across species by carbapenemase genes located on conjugative plasmids. As chromosomal integration of carbapenemase genes has rarely been identified, only a few studies have investigated their advantages to the carbapenem-resistant bacterial community. Here, we confirmed the increased stability of *bla*_IMP-6_ on a chromosome-integrated plasmid in an *Escherichia coli* isolate compared with that on original plasmids in the absence of antibiotic pressure. Although plasmids carrying carbapenemase genes are supposedly lost in successive generations, we found that the complete plasmid backbone was retained in bacterial cells even after the occasional loss of their antibiotic-resistance cassettes. This backbone structure has been observed worldwide to carry various antimicrobial resistance genes. Although the chromosomally integrated plasmid carrying *bla*_IMP-6_ could not be transmitted by conjugation, we found that meropenem treatment for 1 wk allowed the plasmid to be released from the chromosome and spread among *E. coli* strains that were susceptible to meropenem. The copy number of *bla*_IMP-6_ on the plasmid was amplified eight times, resulting in enhanced resistance. Although the carbapenemase producers that carry chromosomal carbapenemase genes comprised of small subpopulations, they functioned as stable, long-term reservoirs of carbapenem resistance that could be disseminated via plasmids with amplified resistance upon meropenem stimulation. Although plasmids occasionally lose their resistance cassettes as a scaffold for the acquisition of another resistance gene, chromosomal integration may contribute to the effective sharing of carbapenem resistance within a population, complicating the development of a strategy to avoid the dissemination of antimicrobial resistance.

**IMPORTANCE:**

Although carbapenem antibiotics are the last resort for combating multidrug-resistant organisms, global dissemination of carbapenem-resistant *Enterobacteriaceae* (CRE) threatens public health. Carbapenemases, which are enzymes responsible for carbapenem resistance, are mainly encoded by genes on plasmids that can be transmitted across bacterial species. Owing to the rarity of chromosomally encoded carbapenemase genes, studies investigating their properties in bacterial communities are lacking. In our study, we revealed the stability of carbapenemase genes on chromosomes compared with those on plasmids, which can be lost through the loss of antimicrobial resistance cassettes despite robust retention of plasmid backbones. Following exposure to meropenem, the carbapenemase gene integrated into the chromosome was released as a plasmid, restarting the dissemination of enhanced carbapenem resistance through amplified copy numbers of carbapenemase genes. Chromosomally encoded carbapenemase genes may function as a reservoir of resistance genes within the bacterial community and challenge infection control against CRE dissemination.

## INTRODUCTION

The emergence and rapid global dissemination of carbapenem-resistant *Enterobacteriaceae* (CRE) threaten the global healthcare system because alternative treatment options are currently limited (1, 2). Carbapenem resistance is primarily conferred by carbapenemases that hydrolyze carbapenem antibiotics (3). Carbapenemase genes are largely plasmid-encoded and are frequently transmitted across species (4, 5). Isolates carrying chromosomally encoded carbapenemase genes are rare, possibly owing to the lack of conjugative transmissibility of these genes (4 - 10). Therefore, only a few studies have investigated the functions of these isolates in carbapenem-resistant bacterial communities (11).

In our previous study, we conducted a plasmidome analysis of 230 CRE isolates carrying *bla*_IMP_ obtained during CRE surveillance in Osaka, Japan (4). During the analysis, we identified an outbreak of *Escherichia coli* isolates carrying IncFIA-type plasmids harboring *bla*_IMP-6_, of which two isolates showed chromosomal integration of the IncFIA-type plasmid through a set of insertion sequences. Thus, in this study, we investigated the characteristics of chromosomally integrated carbapenemase genes in these isolates while focusing on the retention, transmission, and elimination of carbapenemase genes on chromosomes and plasmids.

## MATERIALS AND METHODS

### Study isolates and PFGE analysis

Fifteen *E. coli* isolates carrying *bla*_IMP-6_ on IncFIA-type plasmids and two isolates carrying the IncFIA-type plasmids integrated on their chromosomes were obtained during a previous investigation (4). All isolates were subjected to XbaI-PFGE for phylogenetic analysis and S1-PFGE followed by Southern blotting with a *bla*_IMP-6_ probe for measuring the size of plasmids encoding *bla*_IMP-6_, as previously described (4). Dendrograms were generated from XbaI-PFGE patterns using the unweighted pair group method with arithmetic mean method using BioNumerics software (version 6.6, Applied Maths NV, Sint-Martens-Latem, Belgium). Isolate E301 (E301p) carrying *bla*_IMP-6_ on the IncFIA-type plasmid and isolate E302 (E302c) carrying *bla*_IMP-6_ on its chromosome were used in this study.

### Comparison of *bla*_IMP-6_ elimination

Overnight cultures of isolates E301p and E302c in lysogeny broth (LB) supplemented with meropenem (0.25 µg/mL) were inoculated in LB (3 µL/3 mL) without antibiotics and incubated at 37℃ with shaking. Once a day, 3 µL aliquots of cultures were passaged into 3 mL of LB for 30 days. Cultures were inoculated on MHII agar plates (BD, East Rutherford, NJ, USA), and 100 colonies were reinoculated on MHII agar plates with and without meropenem (1 µg/mL). In cases where colonies did not grow on the plate with meropenem, a loss of *bla*_IMP-6_ was confirmed using PCR analysis targeting *bla*_IMP-6_ of the colony grown on the plate without meropenem. The PCR primers used are listed in Table S3. The proportion of colonies that lost *bla*_IMP-6_ per 100 colonies was calculated. The isolates that lost *bla*_IMP-6_, as well as 10 representative isolates that continued to carry *bla*_IMP-6_, were used for further analysis. The assay was repeated 10 times for each isolate.

### S1-PFGE and whole-genome sequencing

Thirty-nine *E. coli* isolates that lost *bla*_IMP-6_ from plasmid pE301_IMP6 during the 30-day passaging were subjected to S1-PFGE. The size of the plasmids was determined using BioNumerics software (version 6.6; Applied Maths NV, Sint-Martens-Latem, Belgium). Next, DNA fragments separated using S1-PFGE were extracted from the agarose gel with MagExtractor-PCR and Gel Clean up (Toyobo Life Science, Osaka, Japan). Each DNA fragment was subjected to whole-genome sequencing using Illumina MiSeq (Illumina, San Diego, CA, USA) after treatment with reagents in KAPA HyperPlus Library Preparation Kit (Kapa Biosystems, Basel, Switzerland) according to the manufacturer’s instructions. The reads were mapped to the pE301_IMP6 plasmid to determine the genomic structures of *bla*_IMP-6_-negative plasmids using the CLC Genomics Workbench (version 11, Qiagen, Hilden, Germany). The consensus sequences of *bla*_IMP-6_-negative plasmids were compared with the sequence of pE301_IMP6 using EasyFig 2.2.2 (12).

One hundred isolates carrying *bla*_IMP-6_, after 30 days of passage, were subjected to S1-PFGE to determine the size of the plasmid. Based on the results, 20 representative isolates with plasmids of sizes differing from the size of the original plasmid pE301_IMP6 were sequenced using Illumina MiSeq after extracting plasmid DNA fragments from the S1-PFGE gel. The plasmid size of the isolates subjected to sequencing analysis is indicated in Table S1. The sequence reads were compared with the sequence of plasmid pE301_IMP6 using the CLC Genomics Workbench (version 11, Qiagen) to acquire the consensus sequences. The consensus sequence was annotated with RASTtk (13) and was compared with the sequence of pE301_IMP6 using EasyFig 2.2.2 (12).

### Meropenem stimulation and genomic analysis

Overnight culture of isolate E302c in LB was inoculated in 3 mL of LB with or without meropenem (0.25 µg/mL) at 37℃ with shaking. Once a day, 3 µL aliquots of the cultures were collected and passaged for 7 days. After passaging for 7 days, the cultures were incubated overnight in Trypticase Soy broth (BD, NJ, USA) and then subjected to S1-PFGE and Southern blotting analysis with a probe for *bla*_IMP-6_. Briefly, plugs containing DNA were treated with S1 nuclease (Takara Bio, Shiga, Japan). DNA fragments were separated using the CHEF-Mapper XA System (Bio-Rad) in the auto-algorithm mode for 20 hr at 14°C. The DNA fragments were transferred onto a nylon membrane, hybridized with a digoxigenin-labeled probe (Roche, Basel, Switzerland) specific to *bla*_IMP-6_, and detected with CDP-Star Chemiluminescent Substrate (GE Healthcare Life Sciences). The culture after passaging was also inoculated on brain heart infusion agar (BD, NJ, USA), and one colony that was confirmed to carry *bla*_IMP-6_ was stored for subsequent analysis including sequencing. S1-PFGE was performed on the stored strain, and plasmid DNA was extracted from the gel. The plasmid reproduced from the chromosome of isolate E302c via meropenem stimulation was sequenced by Illumina MiSeq (DRR254590). The reads were compared with the chromosomal sequence of isolate E302c using CLC Genomics Workbench (version 11, Qiagen). The consensus sequence was annotated with RASTtk (13) and compared with the chromosomal sequence of E302c using EasyFig 2.2.2 (12). qPCR analysis was conducted to confirm the copy number of *bla*_IMP-6_ on the reproduced plasmid, and the assay was repeated five times as previously described (4).

### Measurement of antimicrobial resistance

Minimum inhibitory concentration of meropenem for isolate E302c after 7 days of passaging with or without meropenem was determined by the broth microdilution method according to the Clinical and Laboratory Standards Institute document M100-S28 (14).

### Measurement of carbapenemase transcription

RT-qPCR analysis was conducted to compare the transcription of *bla*_IMP-6_ with that of *rrsA*. Total RNA was extracted using the RNeasy Mini Kit (Qiagen) after incubation in LB until the optical density at 600 nm (OD_600_) reached 0.4–0.6. RNA was treated with ReverTra Ace qPCR RT Master Mix with gDNA Remover (Toyobo Life Science) to remove contaminating DNA and to reverse-transcribe the RNA to cDNA. *rrsA* encoding the 16S ribosomal RNA served as an endogenous control for normalization. qPCRs were carried out using the Thunderbird Probe and SYBR qPCR Mix (Toyobo Life Science) on a LightCycler 96 System (Roche, Penzberg, Germany). The primers used in this assay are listed in Table S3. qPCR analysis was performed using data from repeated experiments (*n* = 5), and transcript levels were calculated from Ct values using the comparative Ct method (15).

### Measurement of carbapenem hydrolysis

Measurement of carbapenem hydrolysis was conducted as previously described (16). Briefly, the isolates were incubated on MHⅡ agar plates overnight at 37℃, and the colonies were suspended in phosphate-buffered saline at turbidity of 1.0 McFarland standard. A mixture of 50 µL suspension and 50 µL imipenem (50 µg/mL in phosphate-buffered saline) was incubated for 30 min at 37℃. OD_297_ and OD_350_ were measured to calculate the hydrolysis of imipenem. The assay was repeated five times.

### Conjugation

*E. coli* strain TUM2236 (rifampicin-resistant, lactose nonfermenting) (17), used as a donor and E302 isolate (lactose fermenting) after 7 days of passaging with meropenem were incubated overnight on MHII agar plates at 37℃. Colonies from both plates were inoculated together on LB agar plates supplemented with rifampicin (100 µg/mL), meropenem (0.25 µg/mL), and ZnSO_4_ (70 µg/mL). After incubation for 48 hr at 37℃, the colonies were inoculated on a Drigalski lactose agar plate to confirm nonfermentation of lactose by the colony color, and conjugation was confirmed by PCR amplification of *bla*_IMP-6_ from the colony on a Drigalski lactose agar plate as previously described (18).

## RESULTS

### Chromosomally located carbapenemase genes are more stable than plasmid-located genes in the absence of carbapenem

**Fig 1:**
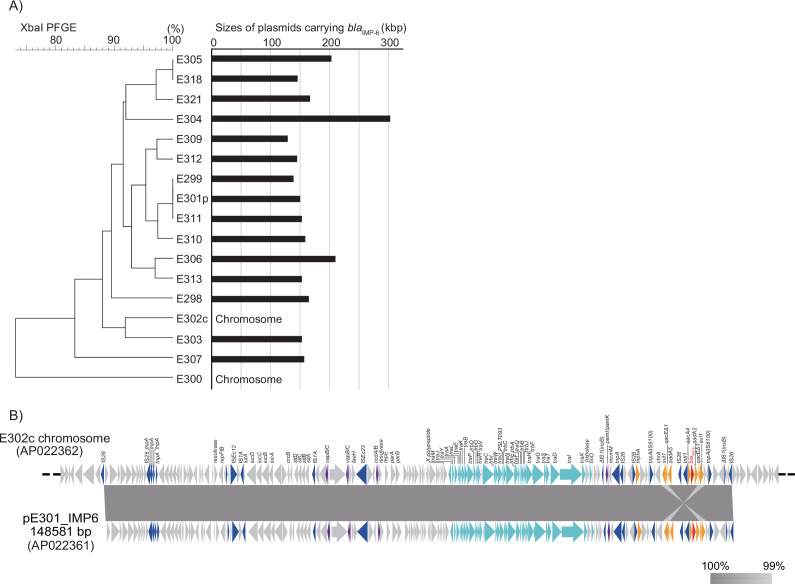
Chromosomal integration of plasmids carrying *bla*_IMP-6_. (**A**) Genetic relationships and sizes of IncF plasmids carrying *bla*_IMP-6_. The study isolates carrying *bla*_IMP-6_ on IncF plasmids, or chromosomally integrated IncF plasmids, were subjected to XbaI-PFGE for phylogenetical analysis. Dendrograms were generated using the UPGMA method. The sizes of the *bla*_IMP-6_-carrier plasmids were determined using S1-PFGE and Southern blotting probing for *bla*_IMP-6_. (**B**) Chromosomal integration of plasmid pE301_IMP6 in isolate E302c. Isolate E302c acquired chromosomal *bla*_IMP-6_ via the incorporation of plasmid pE301_IMP6 bracketed by a set of 26 insertion sequences. The block arrows indicate confirmed or putative open reading frames (ORFs) and their orientations. The arrow size is proportional to the predicted ORF length. The color code is as follows: red, carbapenem-resistance gene; yellow, other antimicrobial resistance genes; light blue, conjugative transfer gene; blue, mobile element; and purple, toxin-antitoxin. Putative, hypothetical, or unknown genes are represented as gray arrows. The gray-shaded area indicates regions with high identity between the sequences. The accession numbers of the plasmids are indicated in brackets.

**Fig 2:**
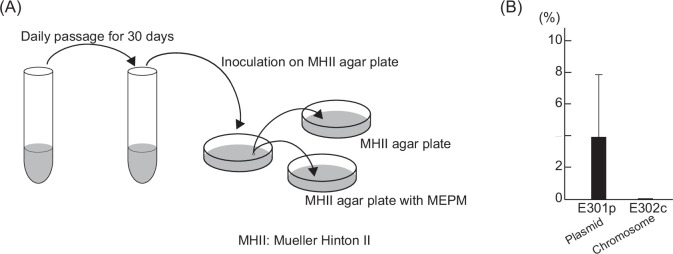
Elimination of *bla*_IMP-6_ on the plasmid and the chromosome after 30 days of passaging without antibiotics. (**A**) Schematic procedure of the experiment. Overnight cultures of isolates E301p and E302c in LB supplemented with meropenem (0.25 µg/mL) were inoculated in LB without antibiotics. After daily passaging for 30 days, the cultures were inoculated on Mueller-Hinton II agar plate, and 100 colonies were reinoculated on agar plates with or without meropenem. The colonies that grew only on the plate without meropenem were subjected to the following analysis after confirming the elimination of *bla*_IMP-6_ by PCR. This procedure was repeated 10 times for each isolate. (**B**) The proportion of *bla*_IMP-6_-eliminated clones after 30 days of passage. *bla*_IMP-6_-eliminated colonies out of the 100 colonies were counted, indicated as percentages. Mean ± standard deviations of the assay repeated 10 times is shown.

Among the 230 CRE isolates obtained in our previous surveillance (4), we identified 15 *E. coli* isolates carrying *bla*_IMP-6_ on IncFIA plasmids, while two *E. coli* isolates integrated the IncFIA into their chromosomes (Fig. 1A). The two isolates seemingly acquired the IncFIA-type plasmid pE301_IMP6 into their chromosomes completely via insertion sequences. Isolate E300 had an inversion of the antibiotic resistance cassette (4), whereas isolate E302c acquired the intact plasmid (Fig. 1B). To compare the stability of *bla*_IMP-6_ located on the plasmid (isolate E301p) and on the chromosome (isolate E302c), we conducted daily subculturing without antibiotics for 30 days and inoculated the isolates on Mueller-Hinton Ⅱ (MHⅡ) agar plates (Fig. 2A). Hundreds of colonies from each subculture were screened 10 times for the presence of *bla*_IMP-6_ (a total of 1,000 colonies for each isolate). *bla*_IMP-6_ in isolate E302c was completely conserved even after 30 days of subculturing, whereas 3.9% of E301p isolates lost *bla*_IMP-6_ after 30 days (Fig. 2B). Thus, carbapenemase genes located on bacterial chromosomes were confirmed to be more stable than those located on plasmids without the selection pressure of carbapenem antibiotics.

### Chromosomally integrated carbapenemase gene excised as a plasmid upon meropenem stimulation

**Fig 3:**
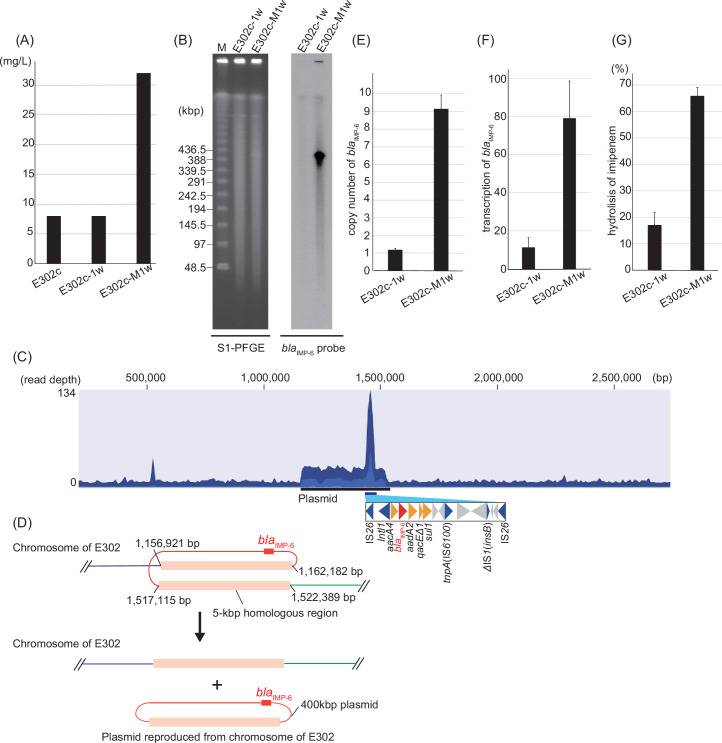
Excision of the plasmid carrying *bla*_IMP-6_ from the chromosome of *Escherichia coli* isolate E302c and enhanced resistance upon meropenem stimulation. (**A**) Minimum inhibitory concentration of meropenem for E302c and E302c after passaging, with or without meropenem. After a week of passaging with or without meropenem, E302c isolates were designated as E302c-M1w and E302c-1w, respectively. (**B**) Southern blotting hybridization with a *bla*_IMP-6_ probe following S1-PFGE of isolate E302c after passaging, with or without meropenem stimulation for a week. The results indicate the excision of approximately 400 kbp long plasmid carrying *bla*_IMP-6_ following meropenem stimulation for a week. (**C**) Mapping of plasmid sequence reads on the chromosomal sequence of isolate E302c. The read depth against the chromosome of E302c is indicated by the depth of the shades, and the three shades of blue, in the order of light to dark, represent minimum, average, and maximum coverage values, respectively, for the aggregated mapped reads. The size of the plasmid determined by mapping is 360 kbp with an additional repeated region carrying *bla*_IMP-6_, corresponding to the result of S1-PFGE. The repeated cassette is enlarged below the figure. Color codes are shown in Fig. 1B. (**D**) The hypothetical mechanism underlying plasmid release. After homologous recombination of a 5 kbp region on the chromosome of E302c, a 400 kbp plasmid carrying *bla*_IMP-6_ seems to be excised from the chromosome. Origins of homologous regions are shown. (**E**) Increased copy numbers of *bla*_IMP-6_ after passaging with meropenem. Copy number of *bla*_IMP-6_ in cells was determined using qPCR with *rrsA* as an internal control (*n* = 5). The formula is 2^-ΔCt^; ΔCt = (Ct *bla*_IMP-6_ – Ct *rrsA*). (**F**) Increased transcription of *bla*_IMP-6_ after passaging with meropenem. Transcription of *bla*_IMP-6_ in cells was determined using qPCR with *rrsA* as an internal control (*n* = 5). The formula is 2^-ΔCt^; ΔCt = (Ct *bla*_IMP-6_ – Ct *rrsA*). (**G**) Increased hydrolysis of imipenem following increased transcription of *bla*_IMP-6_. The percentages of hydrolyzed imipenem within 30 min of incubation with isolate E302 after passaging, with or without meropenem for a week, were measured (*n* = 5). Bars indicate mean ± standard deviation calculated from repeated experiments.

To investigate the effect of meropenem exposure on isolates carrying chromosomally integrated carbapenemase genes, we passaged isolate E302c daily for 7 days with meropenem at a concentration of 0.25 µg/mL. Meropenem resistance in the isolate was enhanced after a week-long exposure to meropenem compared with that in the isolate passaged without meropenem (Fig. 3A). Exposure of E302c to meropenem resulted in the release of a 380 kbp plasmid from the chromosome carrying *bla*_IMP-6_ (Fig. 3B). The DNA fragments of the plasmid extracted from the gel band of S1-nuclease pulsed-field gel electrophoresis (PFGE) were sequenced using Illumina MiSeq, and the sequence was compared with the original chromosomal sequence of isolate E302c. The size of *E. coli* E302c-M1w plasmid was determined to be 360,207 bp, consistent with the result of S1-PFGE (approximately 380 kbp) (Fig. 3B and C). The resultant plasmid was most likely produced via recombination at approximately 5 kbp long homologous regions (Fig. 3D). Notably, the read depth in the excised plasmid mapping the region carrying *bla*_IMP-6_ was deeper than that in other regions (Fig. 3C). qPCR and sequence analysis revealed that the plasmid carried nine copies of *bla*_IMP-6_, which likely occurred through the tandem amplification of the antibiotic resistance cassette sandwiched between a couple of IS*26* genes (Fig. 3C and E). This elevated number led to increased transcription of *bla*_IMP-6_ (Fig. 3F) and thus hydrolysis of imipenem (Fig. 3G), resulting in increased resistance to meropenem. We also confirmed, through PCR of the colony on selective agar, that the released plasmid from the chromosome was conjugatively transferred into another *E. coli* isolate.

### Random deletion on plasmids led to the elimination of carbapenemase genes from the bacterial cells

**Fig 4:**
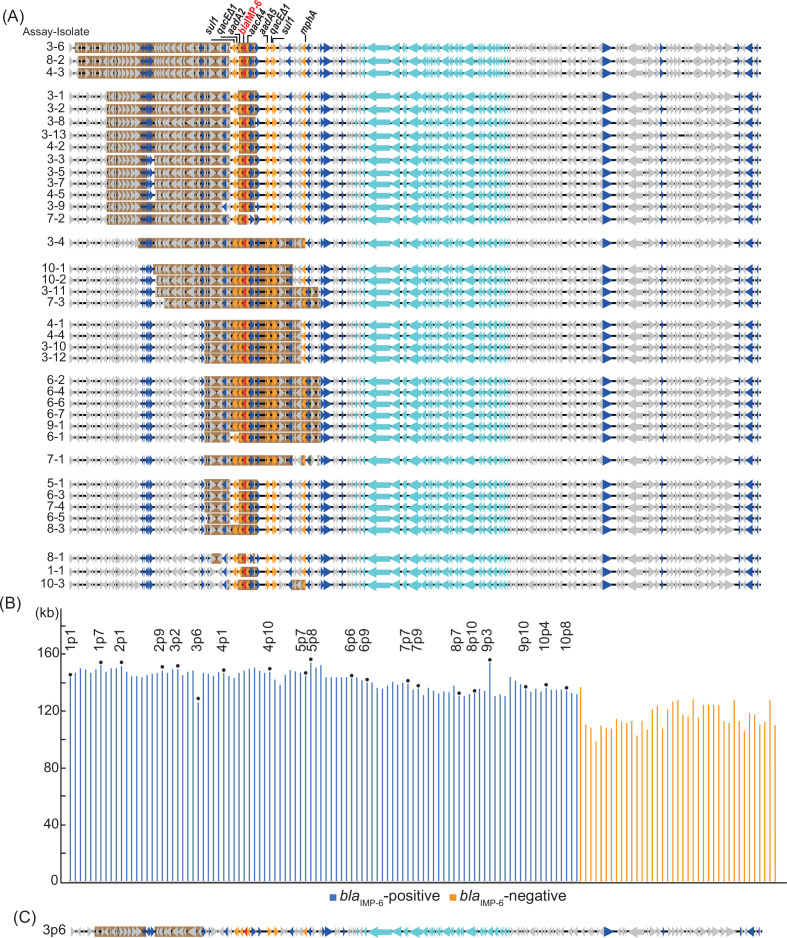
Eliminated region during 30 days of passaging without antibiotic selection. (**A**) Variation in the eliminated region carrying *bla*_IMP-6_ on the plasmid. The names of the isolates are indicated as (assay number–isolate number). Eliminated regions are indicated in the brown layers. The color codes of pE301_IMP6 are as follows: red, carbapenem resistance gene; yellow, other antimicrobial resistance genes; light blue, conjugative transfer gene; and blue, mobile element. Putative, hypothetical, or unknown genes are represented as gray arrows. Among the sequenced isolates that continued to carry *bla*_IMP-6_, isolate 3p6 (No. 6 *bla*_IMP-6_-positive isolate in assay No. 3) was the only one in which a region was eliminated. (**B**) Size of the plasmid after 30 days of passaging without antibiotic pressure selection. The representative 10 isolates from 10 assays (in a total of 100 isolates) and the isolates that lost *bla*_IMP-6_ during passaging were subjected to S1-PFGE to confirm the size of the plasmid. *bla*_IMP-6_-positive plasmids and *bla*_IMP-6_-negative plasmids are indicated as blue and orange bars, respectively. The sequenced isolates that continued carrying *bla*_IMP-6_ are indicated as points with the names of isolates as “assay number p isolate number.” (**C**) Eliminated region on the plasmid that continued carrying *bla*_IMP-6_. Among the sequenced isolates that continued carrying *bla*_IMP-6_, isolate 3p6 (No. 6 *bla*_IMP-6_-positive isolate in assay No. 3) was the only one in which a region was eliminated.

We examined the mechanism underlying the elimination of the carbapenemase genes from the organisms. We identified 39 isolates from the 1,000 isolates obtained following the 30 days successive passaging of isolate E301p that had lost *bla*_IMP-6_. Using S1-PFGE and plasmid genome sequencing, we found that, compared with the original pE301_IMP6 plasmid, all isolates continued to possess the plasmids but lacked the *bla*_IMP-6_ region (Fig. 4A). Among isolates obtained from a single assay, eliminated loci were different from one other, whereas isolates cultured in different assays occasionally carried the plasmids with the same loci eliminated. The eliminated regions were generally limited to the gene cassettes containing antimicrobial resistance genes. Therefore, we speculated that random deletion occurring on the plasmids occasionally leads to the loss of *bla*_IMP-6_.

To confirm the variety of deletions that occurred in the plasmids after 30 days of passage, we conducted S1-PFGE of 100 isolates that carried *bla*_IMP-6_-positive plasmids (10 isolates, each from 10 assays) (Table S1). Compared with that of *bla*_IMP-6_-negative plasmids, the genomic size of *bla*_IMP-6_-positive plasmids was well preserved (Fig. 4B). To investigate the differences in the size of *bla*_IMP-6_-positive plasmids demonstrated by the S1-PFGE results, we conducted whole DNA sequencing of the 20 *bla*_IMP-6_-positive plasmids that were exceptionally large- or small-sized (Fig. 4B) and compared them with the sequence of plasmid pE301_IMP6 (Table S2). Of the 20 plasmids tested, 18 were identical to pE301_IMP6; one plasmid showed a mutation in *pemK* (toxin gene) (19), whereas another exhibited a deletion in the locus similar to the *bla*_IMP-6_-negative plasmids (Fig. 4C). The carbapenemase genes located on the plasmids were lost during the subculturing of the organisms due to the loss of gene cassettes rather than plasmids. The drug-resistance cassettes were more likely to be lost than other regions of the plasmid.

## DISCUSSION

**Fig 5:**
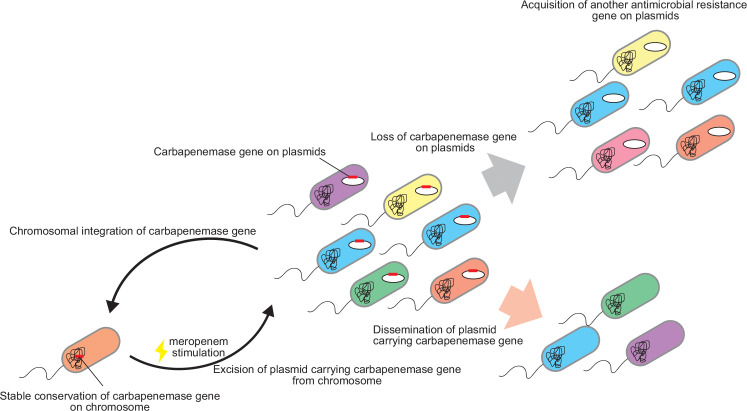
Schematic depiction of carbapenem resistance transmission in a bacterial community.

Bacterial isolates carrying carbapenemase genes on their chromosome are rare (4 - 10). Thus, the functional advantages of chromosomal integration of carbapenemase genes have not been widely investigated. In the present study, we performed a comparative analysis of the stability of chromosomally integrated carbapenemase genes against that of plasmid-encoded carbapenemase genes. After the dissemination of plasmid-encoded carbapenemase genes among other bacterial cells, a small number of the conjugative plasmids were integrated into the chromosome of the host bacterium. Chromosomal carbapenemase genes could be conserved for a prolonged period in bacterial cells as a reservoir of carbapenem resistance, even without antimicrobial selection pressure (Fig. 5).

Once the isolate is stimulated with meropenem, plasmids carrying carbapenemase genes can be released from the chromosome to be transferred to the surrounding carbapenem-susceptible cells. Additionally, carbapenem resistance was highly enhanced by tandem amplification of carbapenemase genes, which is one of the major mechanisms that enhances antimicrobial resistance (4, 20, 21). Although phagemids that are occasionally excised from chromosomes have been reported (22), to the best of our knowledge, this is the first study to show the excision of a conjugative plasmid-encoding carbapenemase from a chromosome upon stimulation with meropenem.

Despite studies on the transmission of carbapenemase genes via conjugative plasmids (4, 5, 23), the mechanism underlying the elimination of carbapenemase genes from CRE isolates remains unclear. We revealed that the IncFIA plasmids containing *bla*_IMP-6_ are not easily eliminated from bacterial cells, although the loci carrying antimicrobial resistance genes were occasionally deleted from the plasmids. The persistence of the pE301_IMP6 plasmid might be related to the toxin-antitoxin system (24). However, the complete stability of all loci in the plasmids other than the antimicrobial resistance cassette and the stability of the entire structure of the *bla*_IMP-6_-positive plasmid indicate the instability of the antimicrobial resistance cassette. Many studies have analyzed the elimination or retention of plasmids carrying antibiotic-resistance genes by investigating the viability of cells on agar plates containing selective antibiotics; however, our results convey the difficulty in estimating the elimination of plasmids (25 - 30). To date, IncF-type plasmids have been frequently reported as transmitters of various β-lactamase genes (31 - 36), as well as the most common replicon-type plasmids encoding carbapenemases (5, 23). A comparison of the DNA structure of different plasmids (Fig. S1) indicated a high similarity among them, implying the function of these plasmids as scaffolds for transmitting various antimicrobial resistance genes without being easily eliminated from bacterial cells.

Despite the clonality of the plasmid pE301_IMP6 and the plasmid integrated into the chromosome of isolate E302c, the locus carrying *bla*_IMP-6_ in pE301_IMP6 was more easily eliminated than that in the chromosome of isolate E302c. Interestingly, most deleted regions only carried the antimicrobial resistance cassette and hypothetical or putative protein genes and were occasionally independent of mobile element genes. Hypothetically, this phenomenon may be due to the difference in stringent regulation of DNA repair between chromosomes and plasmids. For instance, DNA damage-repairing systems, such as the systems involving SulA, which regulate cell-cycle checkpoints by inhibiting cell division upon DNA damage, likely function more stringently for chromosomes than for nonessential plasmids acquired from other microbes (37). Further studies are warranted to provide a better understanding of the stability mechanisms of carbapenemase genes located on chromosomes compared to those located on plasmids. Compared with the locus carrying antimicrobial resistance genes, other loci in the plasmid were suitably conserved, implying an exceptionally high fitness cost to carry an antimicrobial resistance locus without lethal necessity (38, 39). However, contradictory to this hypothesis, the population that lost *bla*_IMP-6_ (4%) was smaller than the population that continued to carry *bla*_IMP-6_ (96%). In addition, the eliminated loci were isolate-specific. If the loss of *bla*_IMP-6_ is due to the release of the locus resulting in high fitness costs, the population that lost *bla*_IMP-6_ should form the majority of the population. If the loss of *bla*_IMP-6_ is because of a deficiency in repairing damaged DNA, *bla*_IMP-6_-negative plasmids may be an unsuccessful subpopulation without clonal proliferation in competition with *bla*_IMP-6_-positive plasmids, suggesting the high stability of the acquired carbapenem resistance once in the host bacterial cell.

In conclusion, we revealed the properties of chromosomally integrated carbapenemase genes in a bacterial community of carbapenemase producers. Carbapenem resistance is mainly transmitted via the conjugation of plasmids carrying carbapenemase genes. These plasmids occasionally lose the carbapenemase genes during passaging without antibiotic selection while retaining the backbone plasmid in the bacterial cells, likely as a scaffold for acquiring resistance genes against other antibiotics. A small population of cells that acquired the plasmid carrying carbapenemase genes integrated the plasmid into their chromosome, utilizing it as a reservoir of carbapenemase genes for the bacterial community. Once exposed to meropenem, these conserved carbapenemase genes on the chromosome are released as plasmids to spread carbapenem resistance among meropenem-susceptible *E. coli* strains. Once acquired, carbapenemase genes are maintained in a bacterial population, with individual subpopulations exhibiting their own advantages, creating a serious challenge in controlling antimicrobial-resistant bacterial infections.

### Limitations of the study

In this study, only a limited variety of *E. coli* strains carrying *bla*_IMP-6_ were studied. A previous study in Thailand observed *Klebsiella pneumoniae* isolates possessing both plasmid-located and chromosomally integrated *bla*_NDM-1_ genes with similar genomic backgrounds (10), suggesting that the similar phenomenon observed in this study may occur in different species carrying different carbapenemase-type genes. Therefore, further studies employing isolates of various backgrounds are necessary. Furthermore, the detailed mechanism underlying the loss of antimicrobial resistance cassettes from plasmids and the excision of carbapenemase gene-carrying plasmid from the chromosome upon meropenem exposure should be investigated.

## Data Availability

Whole-genome sequencing data of isolate E302c have been deposited at the DNA Data Bank of Japan (DDBJ) database and are publicly available as of the date of publication. Accession numbers are listed in Fig. 1. Raw sequence data of plasmid DNA fractions (extracted from the band gels) from E301p cells after 30 days of passaging and those of E302c plasmids isolated from cells after meropenem stimulation for 7 days have been deposited at NCBI. Accession numbers are listed in Table S4. Any additional information required to reanalyze the data reported in this paper is available from the lead contact on request.

## SUPPLEMENTAL MATERIAL

Figure S1 msystems.01275-22-s0001.pdf Comparison of the genomic structure of IncF plasmids carrying various antimicrobial resistance genes. The genomic structure of plasmid pE301_IMP6 was compared with that of the previously reported plasmids identified in BLAST (https://blast.ncbi.nlm.nih.gov/Blast.cgi). The block arrows indicate confirmed or putative ORFs and their orientations. The arrow size is proportional to the predicted ORF length. The color code is as follows: yellow, antimicrobial resistance gene; light blue, conjugative transfer gene; blue, mobile element; and purple, toxin-antitoxin. Putative, hypothetical, or unknown genes are represented as gray arrows. The gray-shaded area indicates regions with high identity between the two sequences. Accession numbers of the plasmids are indicated in brackets.

Table S1 msystems.01275-22-s0002.pdf Sizes of *bla*_IMP-6_-positive or -negative plasmids after 30-day passaging without antibiotics.

Table S2 msystems.01275-22-s0003.pdf Genomic similarity of plasmid pE301_IMP6 and *bla*_IMP-6_-positive plasmids after 30-day passages without antibiotics.

Table S3 msystems.01275-22-s0004.pdf Primers used in this study.

TABLE S4 msystems.01275-22-s0005.pdf Sequence accession numbers of *bla*_IMP-6_-positive or -negative plasmids after 30-day passaging without antibiotics.

ASM does not own the copyrights to Supplemental Material that may be linked to, or accessed through, an article. The authors have granted ASM a non-exclusive, world-wide license to publish the Supplemental Material files. Please contact the corresponding author directly for reuse.
