# Supplementary figures and images for: Carbapenem triggers dissemination of chromosomally integrated carbapenemase genes via conjugative plasmids in Escherichia coli
Source: mSystems. 2023 Jun 5;8(3):e01275-22. doi: 10.1128/msystems.01275-22 (PMC10308940; doi:10.1128/msystems.01275-22)

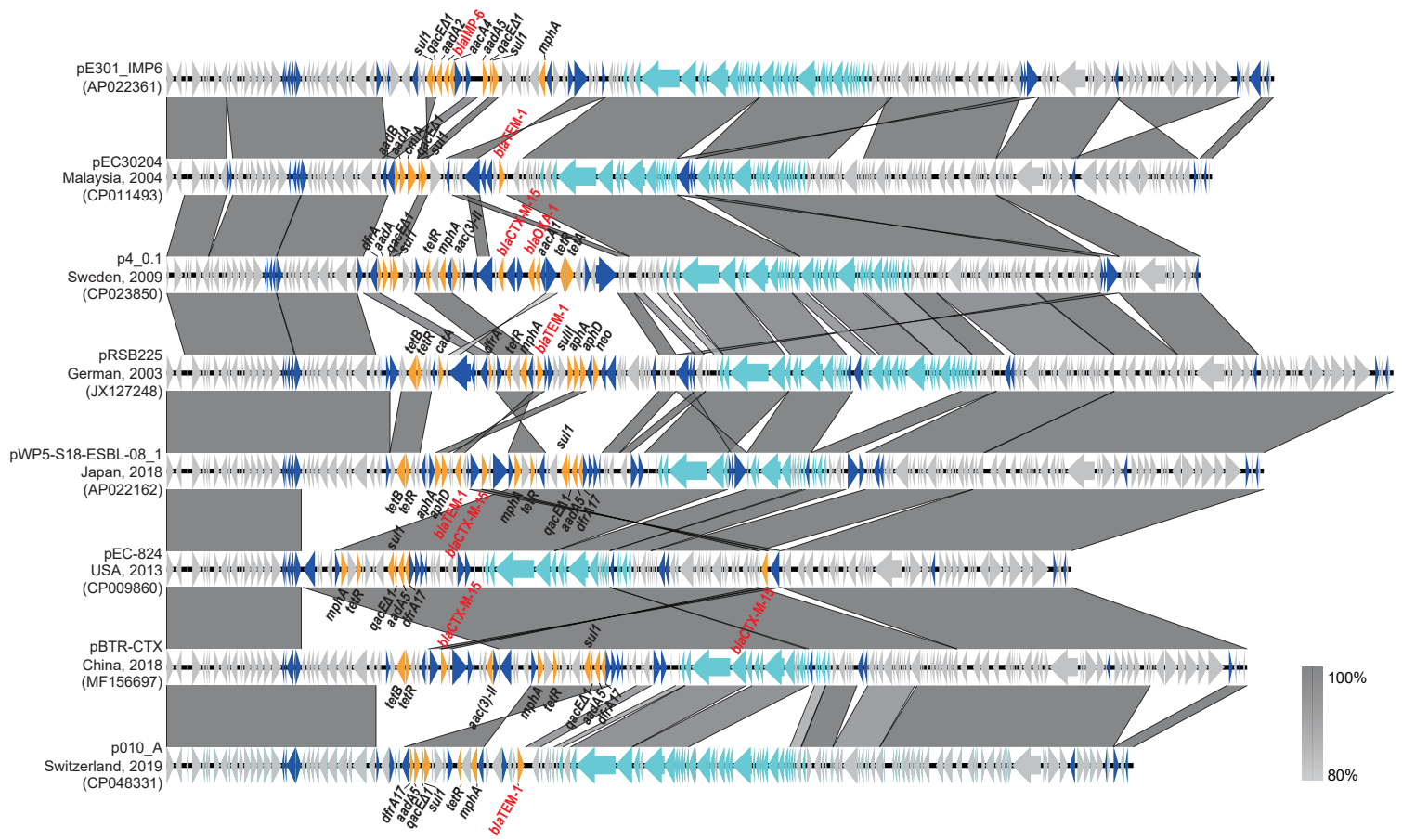

Supplement: Figure S1 — Comparison of the genomic structure of IncF plasmids carrying various antimicrobial resistance genes. The genomic structure of plasmid pE301_IMP6 was compared with that of the previously reported plasmids identified in BLAST (https://blast.ncbi.nlm.nih.gov/Blast.cgi). The block arrows indicate confirmed or putative ORFs and their orientations. The arrow size is proportional to the predicted ORF length. The color code is as follows: yellow, antimicrobial resistance gene; light blue, conjugative transfer gene; blue, mobile element; and purple, toxin-antitoxin. Putative, hypothetical, or unknown genes are represented as gray arrows. The gray-shaded area indicates regions with high identity between the two sequences. Accession numbers of the plasmids are indicated in brackets. [file msystems.01275-22-s0001.pdf]
