# Supplementary material for: Carbapenem triggers dissemination of chromosomally integrated carbapenemase genes via conjugative plasmids in Escherichia coli
Source: mSystems. 2023 Jun 5;8(3):e01275-22. doi: 10.1128/msystems.01275-22 (PMC10308940; doi:10.1128/msystems.01275-22)
Supplement: Table S1 — Sizes of bla IMP-6-positive or -negative plasmids after 30-day passaging without antibiotics. [file msystems.01275-22-s0002.pdf]

Table S1. Sizes of *bla*$_{IMP-6}$-positive or -negative plasmids after 30-day passaging without antibiotics.

bla$_{IMP-6}$-positive	Plasmid Size	*bla*$_{IMP-6}$-positive	Plasmid Size	*bla*$_{IMP-6}$-positive	Plasmid Size	*bla*$_{IMP-6}$-negative	Plasmid Size
1P1	143.95	4P9	147.02	8P7	130.66	1-1	136.92
1P2	147.04	4P10	147.79	8P8	130.66	3-1	110.42
1P3	150.18	5P1	142.28	8P9	131.97	3-2	108.46
1P4	149.38	5P2	138.51	8P10	132.63	3-3	99.06
1P5	147.04	5P3	145.58	9P1	135.83	3-4	109.87
1P6	149.38	5P4	148.96	9P2	134.38	3-5	108.46
1P7	152.55	5P5	148.09	9P3	154.27	3-6	107.8
1P8	147.82	5P6	147.23	9P4	130.66	3-7	114.63
1P9	150.18	5P7	144.83	9P5	131.97	3-8	112.64
1P10	150.18	5P8	154.27	9P6	130.66	3-9	111.66
2P1	151.75	5P9	150.7	9P7	144.15	3-10	113.31
2P2	147.82	5P10	152.45	9P8	141.56	3-11	102.92
2P3	144.72	6P1	143.95	9P9	138.99	3-12	113.31
2P4	144.72	6P2	143.95	9P10	135.19	3-13	107.18
2P5	143.95	6P3	143.95	10P1	133.66	4-1	121.65
2P6	145.49	6P4	143.95	10P2	135.83	4-2	123.6
2P7	146.27	6P5	143.95	10P3	133.94	4-3	107.87
2P8	147.04	6P6	143.21	10P4	136.46	4-4	121.65
2P9	148.6	6P7	143.95	10P5	135.19	4-5	126.73
2P10	147.02	6P8	141.71	10P6	135.19	5-1	127.77
3P1	149.33	6P9	140.22	10P7	135.19	6-1	117.55
3P2	149.33	6P10	140.22	10P8	134.56	6-2	116.28
3P3	145.52	7P1	136.55	10P9	132.69	6-3	128.34
3P4	147.79	7P2	135.83	10P10	132.06	6-4	115.65
3P5	148.56	7P3	138.01			6-5	124.57
3P6	126.27	7P4	140.65			6-6	124.57
3P7	147.02	7P5	138.61			6-7	124.57
3P8	146.25	7P6	139.97			7-1	123.94
3P9	144.8	7P7	139.29			7-2	113.04
3P10	147.79	7P8	135.27			7-3	111.73
4P1	147.02	7P9	135.93			7-4	127.7
4P2	144.8	7P10	131.31			8-1	113.04
4P3	143.41	8P1	136.55			8-2	105.81
4P4	147.02	8P2	134.38			8-3	118.83
4P5	148.56	8P3	132.63			9-1	117.55
4P6	149.91	8P4	133.95			10-1	110.57
4P7	150.65	8P5	133.29			10-2	112.49
4P8	148.43	8P6	137.95			10-3	127.7

The names of the isolates are indicated as (assay number P isolate number) for *bla*$_{IMP-6}$ positives, and (assay number - isolate number) for *bla*$_{IMP-6}$ negatives. The sequenced isolates were indicated in red letters.
