# Supplementary material for: Carbapenem triggers dissemination of chromosomally integrated carbapenemase genes via conjugative plasmids in Escherichia coli
Source: mSystems. 2023 Jun 5;8(3):e01275-22. doi: 10.1128/msystems.01275-22 (PMC10308940; doi:10.1128/msystems.01275-22)
Supplement: Table S2 — Genomic similarity of plasmid pE301_IMP6 and bla IMP-6-positive plasmids after 30-day passages without antibiotics. [file msystems.01275-22-s0003.pdf]

Table S2. Genomic similarity of plasmid pE301_IMP6 and *bla*$_{IMP-6}$-positive plasmids after 30-day passages without antibiotics.

Isolate	Coverage (%)	Identity (%)
1p1	100	100
1p7	100	100
2p1	100	100
2p9	100	100
3p2	100	100
3p6	86	100
4p1	99	99.99
4p10	100	100
5p7	100	100
5p8	100	100
6p6	100	100
6p9	100	100
7p7	100	100
7p9	100	100
8p10	100	100
8p7	100	100
9p10	100	100
9p3	100	100
10p4	100	100
10p8	100	100
