# Supplementary material for: Carbapenem triggers dissemination of chromosomally integrated carbapenemase genes via conjugative plasmids in Escherichia coli
Source: mSystems. 2023 Jun 5;8(3):e01275-22. doi: 10.1128/msystems.01275-22 (PMC10308940; doi:10.1128/msystems.01275-22)
Supplement: Table S3 — Primers used in this study. [file msystems.01275-22-s0004.pdf]

Table S3. Primers used in this study.

Primer name	Primer sequence (5'' to 3'')	
**PCR analysis for confirmation of the presence of *bla*$_{IMP-6}$**		
IMPF	GCTACCGCAGCAGAGTCTTT	
IMPR	CAAGAGTGATGCGTCTCCAA	
**Southern hybridization of *bla*$_{IMP-6}$**		
IMPF	GCTACCGCAGCAGAGTCTTT	
IMPR	CAAGAGTGATGCGTCTCCAA	
**qPCR for analysis of transcription of *bla*$_{IMP-6}$**		
IMP6-qPCRF	GGTTTAGGCAATTTGGGTGA	*bla*$_{IMP-6}$ transcription
IMP6-qPCRR	CAAGAGTGATGCGTCTCCAA	
rrsA-F	GTGGCTACGATTGCATTCCA	Internal control with *rrsA* on chromosome
rrsA-R	TAGCGCATTAATTACGCCAA	
