# Supplementary material for: Carbapenem triggers dissemination of chromosomally integrated carbapenemase genes via conjugative plasmids in Escherichia coli
Source: mSystems. 2023 Jun 5;8(3):e01275-22. doi: 10.1128/msystems.01275-22 (PMC10308940; doi:10.1128/msystems.01275-22)
Supplement: TABLE S4 — Sequence accession numbers of bla IMP-6-positive or -negative plasmids after 30-day passaging without antibiotics. [file msystems.01275-22-s0005.pdf]

Table S4. Sequence accession numbers of *bla*$_{IMP-6}$-positive or -negative plasmids after 30-day passaging without antibiotics.

bla$_{IMP-6}$-positive	Accession number	*bla*$_{IMP-6}$-negative	Accession number
1p1	DRX242034	1-1	DRX241998
1p7	DRX242035	3-1	DRX241999
2p1	DRX242036	3-2	DRX242004
2p9	DRX242037	3-3	DRX242006
3p2	DRX242038	3-4	DRX242007
3p6	DRX242039	3-5	DRX242008
4p1	DRX242040	3-6	DRX242009
4p10	DRX242041	3-7	DRX242010
5p7	DRX242042	3-8	DRX242011
5p8	DRX242043	3-9	DRX242012
6p6	DRX244307	3-10	DRX242000
6p9	DRX242044	3-11	DRX242001
7p7	DRX242045	3-12	DRX242002
7p9	DRX242046	3-13	DRX242003
8p10	DRX242047	3-14	DRX242005
8p7	DRX242048	4-1	DRX242013
9p10	DRX242049	4-2	DRX242014
9p3	DRX242050	4-3	DRX242015
10p4	DRX242051	4-4	DRX242016
10p8	DRX242052	4-5	DRX242017
		5-1	DRX242018
		6-1	DRX242019
		6-2	DRX242020
		6-3	DRX242021
		6-4	DRX242022
		6-5	DRX242023
		6-6	DRX242024
		6-7	DRX242025
		7-1	DRX242026
		7-2	DRX242027
		7-3	DRX242028
		7-4	DRX242029
		8-1	DRX242030
		8-2	DRX242031
		8-3	DRX242032
		9-1	DRX242033
		10-1	DRX264854
		10-2	DRX244305
		10-3	DRX244306
